# Significant response to atezolizumab plus bevacizumab treatment in unresectable hepatocellular carcinoma with major portal vein tumor thrombus: a case report

**DOI:** 10.1186/s12876-021-02053-4

**Published:** 2021-12-15

**Authors:** Shohei Komatsu, Yoshimi Fujishima, Masahiro Kido, Kaori Kuramitsu, Tadahiro Goto, Hiroaki Yanagimoto, Hirochika Toyama, Takumi Fukumoto

**Affiliations:** 1 Division of Hepato-Biliary-Pancreatic Surgery, Department of Surgery, Kobe University Graduate School of Medicine, 7-5-2 Kusunoki-cho, Chuo-ku, Kobe, Hyogo 650-0017 Japan; 2 Division of Medical Oncology, Kobe Minimally Invasive Cancer Center, Kobe, Japan

**Keywords:** Hepatocellular carcinoma, Atezolizumab plus bevacizumab, Portal vein tumor thrombus, Case report

## Abstract

**Background:**

Hepatocellular carcinoma (HCC) with major portal vein tumor thrombus (Vp4 PVTT) is an extremely advanced tumor with limited treatment options. Systemic chemotherapy is the only recommended treatment option, and atezolizumab plus bevacizumab has recently emerged as a first-line treatment option.

**Case presentation:**

We describe the case of an 82-year-old man with unresectable advanced HCC with Vp4 PVTT who achieved a significant response to atezolizumab plus bevacizumab treatment. A single administration of atezolizumab plus bevacizumab ensured significant anti-tumor effects (regression in the tumor size and PVTT, portal vein recanalization, and serum alfa-fetoprotein levels decreased from 90,770 to 89 ng/mL). The patient continued with atezolizumab monotherapy, and after nine consecutive regimens, there was no apparent sign of residual tumor.

**Conclusions:**

This case demonstrates the powerful anti-tumor effect of atezolizumab plus bevacizumab treatment for advanced HCC with Vp4 PVTT, suggesting that these agents can be a promising treatment option for such refractory tumors.

## Background

Hepatocellular carcinoma (HCC) with major portal vein tumor thrombus (PVTT) is an extremely advanced-stage tumor with limited treatment options [1]. Especially after invading into the main or contralateral portal trunk, HCC with PVTT is associated with poor prognosis because of the underlying intra-hepatic metastasis through the portal vein and the deterioration of liver function caused by decreased portal blood flow. Current guidelines recommend systemic chemotherapy for such advanced tumors, with sorafenib being the only first-line treatment for unresectable HCC since its approval in 2007. In the recent REFLECT trial, lenvatinib showed non-inferiority to sorafenib in terms of overall survival [2]. The introduction of cell-based immunotherapy, including dendric cells and cytokine-induced killer cells, may bring effective benefits to patients with HCC [3]. Recently, immune checkpoint blockade therapies targeting programmed cell death protein 1, programmed death ligand 1, and cytotoxic T lymphocyte antigen 4 have been shown to enhance anti-tumor immune responses and exhibited great potential in HCC therapy [4]. The combined use of atezolizumab plus bevacizumab in the IMbrave 150 trial demonstrated superior progression-free and overall survival than that associated with sorafenib [5]. Accordingly, atezolizumab plus bevacizumab has become a first-line HCC treatment. Here, we report a case of HCC with PVTT invasion from the ipsilateral to the main and contralateral portal trunk, which showed a significant response to atezolizumab plus bevacizumab treatment.

## Case presentation

**Fig. 1:**
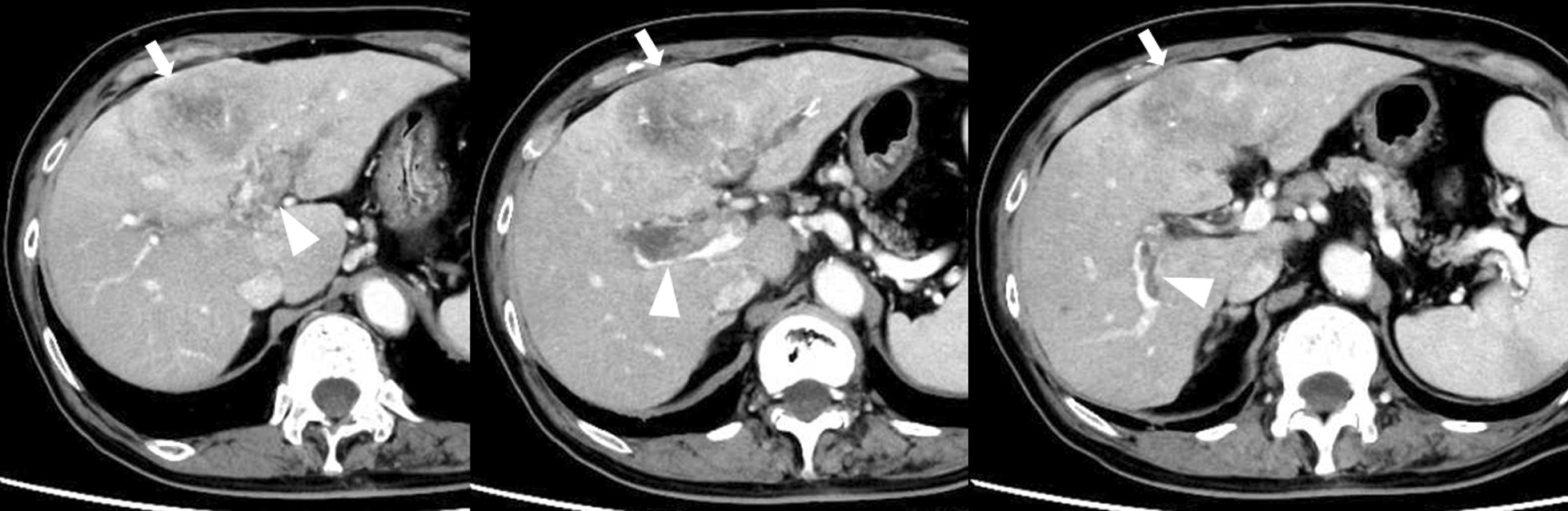
Abdominal computed tomography revealing a huge hepatocellular carcinoma located at segment 4 (white arrow). The portal vein tumor thrombus has spread from the adjacent portal vein to the left portal trunk, main portal trunk, through to the contralateral right portal trunk (arrowhead). The tip of the portal vein tumor thrombus has invaded into the bifurcation of the anterior and posterior branches; the anterior branch is filled with tumor thrombus, while the posterior branch is filled with tumors or blood thrombi

**Fig. 2:**
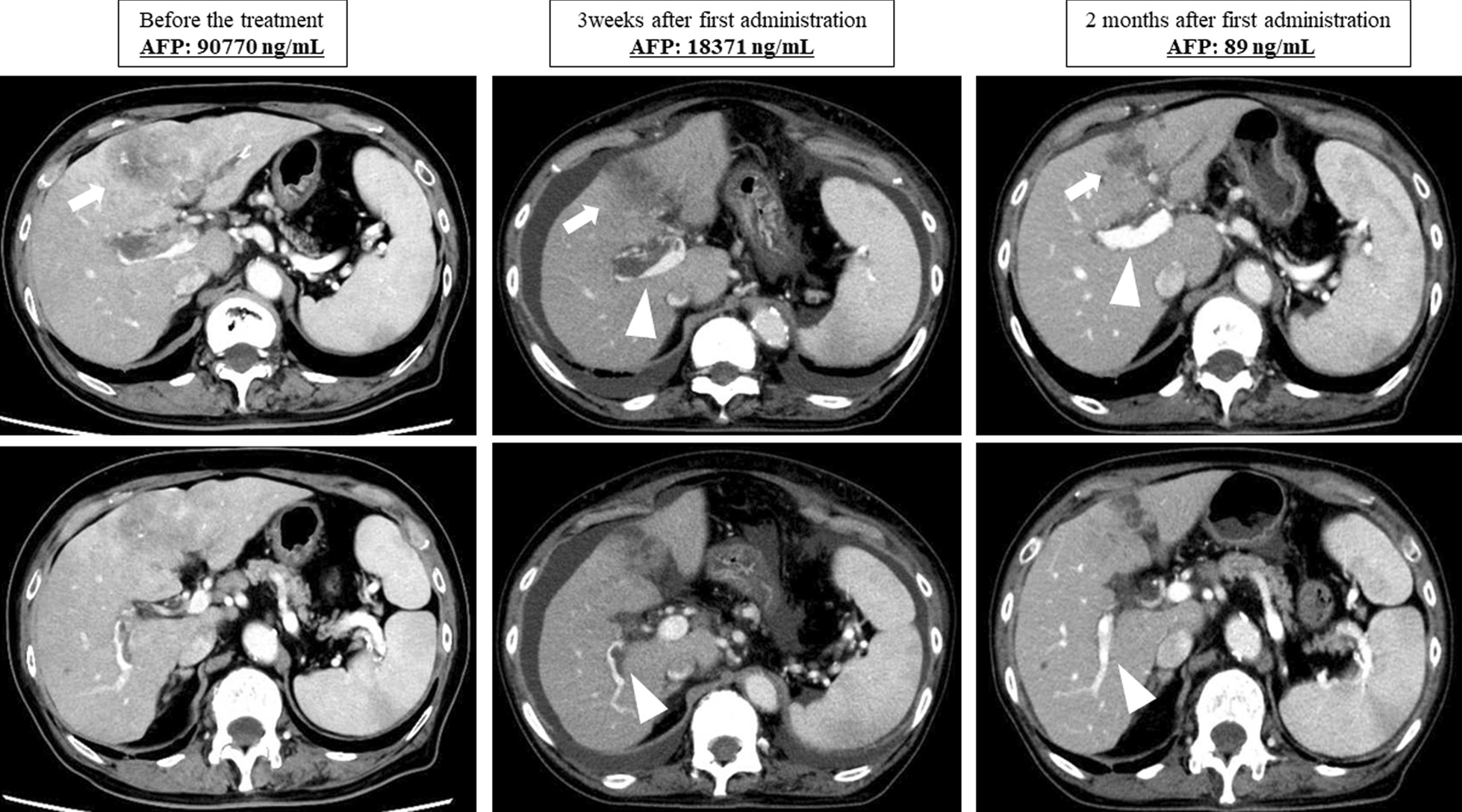
Abdominal computed tomography showing size reduction of the main tumor and the portal vein tumor thrombus (white arrow). The tip of the portal vein tumor thrombus has regressed from the bifurcation of the anterior and posterior branches to the left trunk, and the right portal vein is recanalized. Serum alfa-fetoprotein level decreased from 90,700 ng/mL before the treatment to 18,371 ng/mL (3 weeks later) and 89 ng/mL (2 months later). AFP, alfa-fetoprotein

**Fig. 3:**
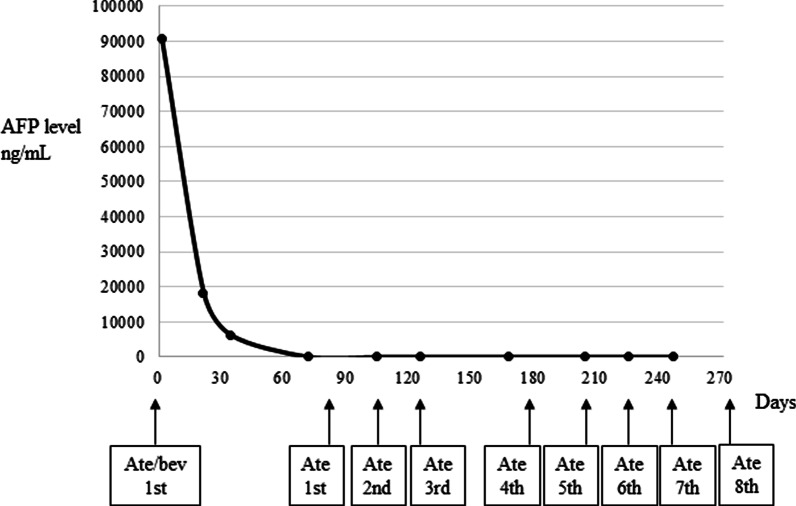
Transition of the serum alfa-fetoprotein level during the treatment course. AFP, alfa-fetoprotein; Ate/bev, atezolizumab plus bevacizumab treatment; Ate, atezolizumab treatment

An 82-year-old male patient with angina on anticoagulant medication and without viral infection was referred to our department for the surgical treatment of HCC. His personal and family medical history was otherwise unremarkable. Abdominal computed tomography (CT) showed a huge HCC mass in segment 4 and PVTT invasion from the nearby portal vein to the left portal trunk, main portal trunk, through to the contralateral right portal trunk. The tip of the PVTT progressed over to the bifurcation of the anterior and posterior branches of the portal trunk. The anterior branch was filled with PVTT, while the posterior branch was filled with PVTT or blood thrombus (Fig. 1). No apparent intra- and extra-hepatic metastases were detected other than the main tumor. Laboratory data showed a serum albumin level of 3.8 g/dL, total bilirubin level of 0.5 mg/dL, platelet count of 13.9 × 10^4^/uL, and a Child-Pugh score of 6. Serum levels of alfa-fetoprotein (AFP) and protein induced by vitamin K absence or antagonist II were 90,770 ng/mL and 2847 mAU/mL, respectively. An antithrombotic drug was administered for the PVTT. Due to the overwhelming PVTT extensions, poor performance status, and old age, the patient was deemed not to have a surgical indication. Therefore, a combination treatment using atezolizumab plus bevacizumab with radiotherapy for PVTT was selected. During preparation for radiotherapy, one-time atezolizumab (1200 mg) and bevacizumab (15 mg/kg) were administered. He developed anal pain and persistent fever 9 days after administration, and CT showed perianal abscess due to anal fistula. Although he recovered soon after percutaneous abscess drainage, this adverse event interrupted atezolizumab plus bevacizumab treatment, and radiotherapy could not be introduced. Abdominal CT, conducted 3 weeks after the first administration, showed size reduction of the main tumor and PVTT, with a reduction in tumor enhancement on contrast-enhanced CT. Serum AFP decreased from 90,700 ng/mL before treatment to 18,371 ng/mL and 6301 ng/mL 3 and 5 weeks after atezolizumab plus bevacizumab treatment, respectively. Considering the excellent response to atezolizumab plus bevacizumab treatment (one-time administration), we decided to continue with this treatment after creating colostomy. Colostomy was performed 47 days after the first administration of atezolizumab plus bevacizumab. Subsequently, beginning from the 83rd day after the first administration of atezolizumab plus bevacizumab, atezolizumab monotherapy was initiated. The reason for choosing atezolizumab monotherapy was that the bleeding from the colostomy persists from the collateral blood circulation due to the liver cirrhosis. The serum AFP decreased to 89 ng/mL just before the next atezolizumab monotherapy, and abdominal CT showed continued partial response (Fig. 2); serum AFP levels kept decreasing toward the normal range (Fig. 3). He received a one-time atezolizumab plus bevacizumab treatment and continued with atezolizumab monotherapy afterward (9 regimens administered until now); there is no apparent sign of residual tumors at 9 months after the introduction of atezolizumab plus bevacizumab treatment.

## Discussion and conclusion

We report a case of HCC with PVTT invasion into the contralateral portal trunk (Vp4 PVTT, a classification of PVTT according to the Liver Cancer Study Group of Japan) [6], which responded excellently to atezolizumab plus bevacizumab treatment. Tumor size and invasion were significantly reduced and weakened, and tumor progression was controlled with the remarkable regression of PVTT and reduction of serum AFP levels after one-time atezolizumab plus bevacizumab administration. To the best of our knowledge, this case is the first to show a tremendous treatment response of HCC with Vp4 PVTT to atezolizumab plus bevacizumab treatment, indicating the strong potential of this treatment.

In this case, we could not perform radiotherapy or continue chemotherapy as the adverse event occurred because of atezolizumab plus bevacizumab treatment. The duration between the first and the second chemotherapy regimens (without bevacizumab) was 83 days; thus, the drastic tumor response observed before the second chemotherapy was completely induced by the one-time atezolizumab plus bevacizumab treatment (Fig. 2). Antithrombotic treatment was continued because the effectiveness of antithrombotic treatment for HCC with PVTT has been reported in literature [7]. The tip of the PVTT regressed from the bifurcation of the anterior and posterior branches to the left trunk, and tumor enhancement completely disappeared without any sign of tumor viability. The clinical course of this case is surprising because of the powerful potential of atezolizumab plus bevacizumab treatment for HCC with advanced PVTT.

After the results of the SHARP and Asia-Pacific trials were published [8, 9], patients with HCC and macroscopic PVTT have been treated with sorafenib worldwide with hopes of good survival outcomes. However, HCC patients with PVTT have been historically categorized as a subgroup of patients with “macroscopic vascular invasion” of which there are no stratified subgroup analyses [10, 11]. Among tumors with PVTT, Vp3 and Vp4 (Vp3/4) PVTT (Vp3: PVTT with the tip of the thrombus in the ipsilateral first branch, and Vp4: PVTT with the tip of the thrombus reaching either the portal trunk or more distal contralateral portal branch), classified according to the PVTT classification by the Liver Cancer Study Group of Japan [6] should be considered as clinical urgencies that can become fatal in just 2 weeks. This is because the time required for PVTT progression from the ipsilateral first portal branch to the portal trunk (Vp3 to Vp4) was reported to be just 11.5 days [12]. The reported median survival time associated with sorafenib for HCC patients with Vp3/4 PVTT is only 3–4 months [13, 14].

With the emergence of lenvatinib and atezolizumab plus bevacizumab treatment, the chemotherapeutic strategy for unresectable HCC has changed in this multimodal chemotherapy era [2, 5]. The objective response rate and disease control rate (defined by the modified Response Evaluation Criteria in Solid Tumors) of lenvatinib in the REFLECT trial were 40.6% and 73.8%, respectively [2], and those of atezolizumab plus bevacizumab treatment in the IMbrave150 trial were 33.2% and 72.3%, respectively [5]. The IMbrave150 included high-risk patients, defined as those with Vp4 PVTT, tumors occupying more than 50% of the liver, and bile-duct invasion. The median survival time of patients with a high-risk status treated with atezolizumab plus bevacizumab was reported recently to be only 7.6 months, whereas that of those with a non-high-risk status was 22.8 months [15].

Considering the relatively low objective response rate and limited survival impact even with lenvatinib and atezolizumab plus bevacizumab treatment for highly advanced HCC, the therapeutic effects of these modalities appear to be limited. HCC with Vp3/4 PVTT-associated death is mainly attributable to PVTT extension-induced liver failure [16]. Therefore, the further extension of PVTT should be controlled to secure the therapeutic time window while managing such high-risk HCC cases. Monotherapy with chemotherapy may be insufficient to prevent further PVTT progression; thus, introducing localized treatment with hepatectomy [17, 18] or radiotherapy [19, 20] becomes essential clinically as a management strategy. Most importantly, this case demonstrated the powerful potential of one-time atezolizumab plus bevacizumab administration that induced a tremendous tumor response. The novelty of this case should be recognized, and its potential as promising treatment option for HCC with Vp3/4 PVTT needs more attention.

The introduction of immune-checkpoint inhibitors has led to a paradigmatic change in the systemic treatment of HCC. Several promising phase 3 trials have been ongoing based on the favorable outcomes of phase 1/2 [4], and HCC treatment is now entering a new era with immunotherapy. Further analysis regarding the treatment outcomes for patients with HCC and severe vascular invasion will be warranted. Along with the development of analysis regarding the prediction of treatment effectiveness before the introduction of chemotherapy, more sophisticated tailor-made treatments that involve selecting the population of patients who can obtain the benefit from systemic treatment may emerge in future.

In conclusion, we encountered a case of HCC with Vp4 PVTT treated with atezolizumab plus bevacizumab treatment, which showed significant response and no sign of residual tumor viability. Considering the limited treatment options and poor survival associated with HCC with Vp3/4 PVTT, the present case findings present significant impact for selecting treatment modalities.

## Data Availability

Data will be provided upon reasonable request and in concordance with maximal protection of the patient privacy.

## Abbreviations

HCC: Hepatocellular carcinoma
PVTT: Portal vein tumor thrombus
CT: Computed tomography
AFP: Alfa-fetoprotein
